# Cerebral small vessel disease combined with cerebral collaterals to predict the prognosis of patients with acute large artery atherosclerotic stroke

**DOI:** 10.3389/fneur.2022.969637

**Published:** 2022-08-11

**Authors:** Cunsheng Wei, Tingwen Shen, Xuelian Tang, Yuanyuan Gao, Xiaorong Yu, Xuemei Chen

**Affiliations:** ^1^Department of Neurology, The Affiliated Jiangning Hospital With Nanjing Medical University, Nanjing, China; ^2^The Health Promotion Center, The First Affiliated Hospital of Nanjing Medical University, Nanjing, China; ^3^Department of General Practice, The Affiliated Jiangning Hospital With Nanjing Medical University, Nanjing, China

**Keywords:** collaterals, cerebral small vessel disease, prognosis, large artery, stroke

## Abstract

**Background and purpose:**

Besides cerebral collaterals, few studies have examined other additional factors affecting the prognosis of patients with large artery atherosclerotic (LAA) stroke. Our study aims to explore the effect of the cerebral small vessel disease (SVD) and the effects of its interaction with cerebral collaterals on the prognosis of patients with acute LAA stroke.

**Method:**

Patients aged 18 years or older with LAA stroke within 24 h after stroke onset were consecutively enrolled. The functional outcome was determined using the modified Rankin Scale (mRS) at 3 months after stroke onset. Logistic multivariate analyses were used to identify the risk factors for stroke prognosis. Receiver operating characteristic (ROC) curves were constructed to compare the effects of cerebral collaterals and SVD on predicting the prognosis.

**Results:**

Of the 274 enrolled patients, 174 (63.50%) were identified as having a favorable prognosis, and 100 (36.50%) were identified as having an unfavorable prognosis. After adjusting for covariates, the logistic regression analysis identified that unfavorable prognosis was related to the total SVD score (Model 1, adjusted odds ratio = 1.73, 95% CI: 1.15–2.61, *P* < 0.01; Model 2, adjusted odds ratio = 1.85, 95% CI: 1.23–2.79, *P* < 0.01) and Tan score (Model 1, adjusted odds ratio = 0.38, 95% CI: 0.23–0.64, *P* < 0.01; Model 2, adjusted odds ratio = 0.52, 95% CI: 0.33–0.82, *P* < 0.01). Compared with cerebral collaterals (AUC = 0.59; 95% CI: 0.52–0.67; *P* < 0.01) or SVD (AUC = 0.62; 95% CI: 0.56–0.69; *P* < 0.01) alone, the combination of collaterals and SVD (AUC = 0.66; 95% CI: 0.59–0.73; *P* < 0.01) had higher diagnostic value for an unfavorable prognosis, and the optimal sensitivity and specificity were 77.01 and 53.00%, respectively.

**Conclusions:**

The total SVD burden was related to the prognosis of patients with LAA stroke. Compared with cerebral collaterals or SVD alone, cerebral collaterals combined with total SVD burden are better at predicting the prognosis of patients with acute LAA stroke.

## Introduction

Large artery atherosclerosis is responsible for ~17% of all cases of ischemic stroke (1) and is considered a systemic disease that may lead to both cardiovascular and cerebrovascular diseases (2). It is an important cause of global disability and death in patients with large artery atherosclerotic (LAA) stroke, despite considerable progress in the treatment of acute stroke with intravenous thrombolysis (IVT) and mechanical thrombectomy (MT) (3, 4). Several risk factors related to the prognosis of LAA stroke, including traditional risk factors (such as age, hypertension, diabetes, hyperlipidemia, smoking, etc.), and the role of cerebral collaterals has been the focus of research recently (5). A retrospective study from two comprehensive stroke centers indicated that collaterals predict patient outcomes, regardless of the time last known to be normal in patients with LAA stroke who were treated with MT (6). Moreover, in patients with ischemic stroke caused by occlusion of a proximal intracranial artery who were treated with EVT, higher collateral scores are associated with a better functional outcome (7).

Recently, with the development of neuroimaging, new neuroimaging markers of cerebral small vessel disease (SVD) related to predicting the prognosis of LAA stroke have attracted increasing attention. Markers of SVD on magnetic resonance imaging (MRI) include white matter hyperintensities (WMH), lacunes, cerebral microbleeds (CMBs) and enlarged perivascular spaces (EPVS). Some studies have indicated that cerebral SVD is potentially related to ischemic stroke (8–12); However, researchers have not determined whether cerebral SVD increases the risk of a poor prognosis for LAA stroke and few studies have explored the combined effect of cerebral collaterals and SVD on the prognosis. Therefore, our study aims to explore the effect of the SVD burden and its combined effects with cerebral collaterals on the prognosis of acute LAA stroke to provide more guidance for clinical decisions.

## Methods

### Data source

Patients were screened at the Affiliated Jiangning Hospital with Nanjing Medical University. The study was approved by the hospital ethics committee. Patients aged 18 years or older with acute ischemic stroke within 24 h after stroke onset were consecutively enrolled from 9 January 2019, to 21 December 2021. The inpatient medical record system contains data on patient demographics, clinical and imaging features and treatment details. Data on patient demographics, clinical history, clinical presentation, laboratory results, treatment, follow-up examinations and outcome were collected.

### Study design and population

**Figure 1:**
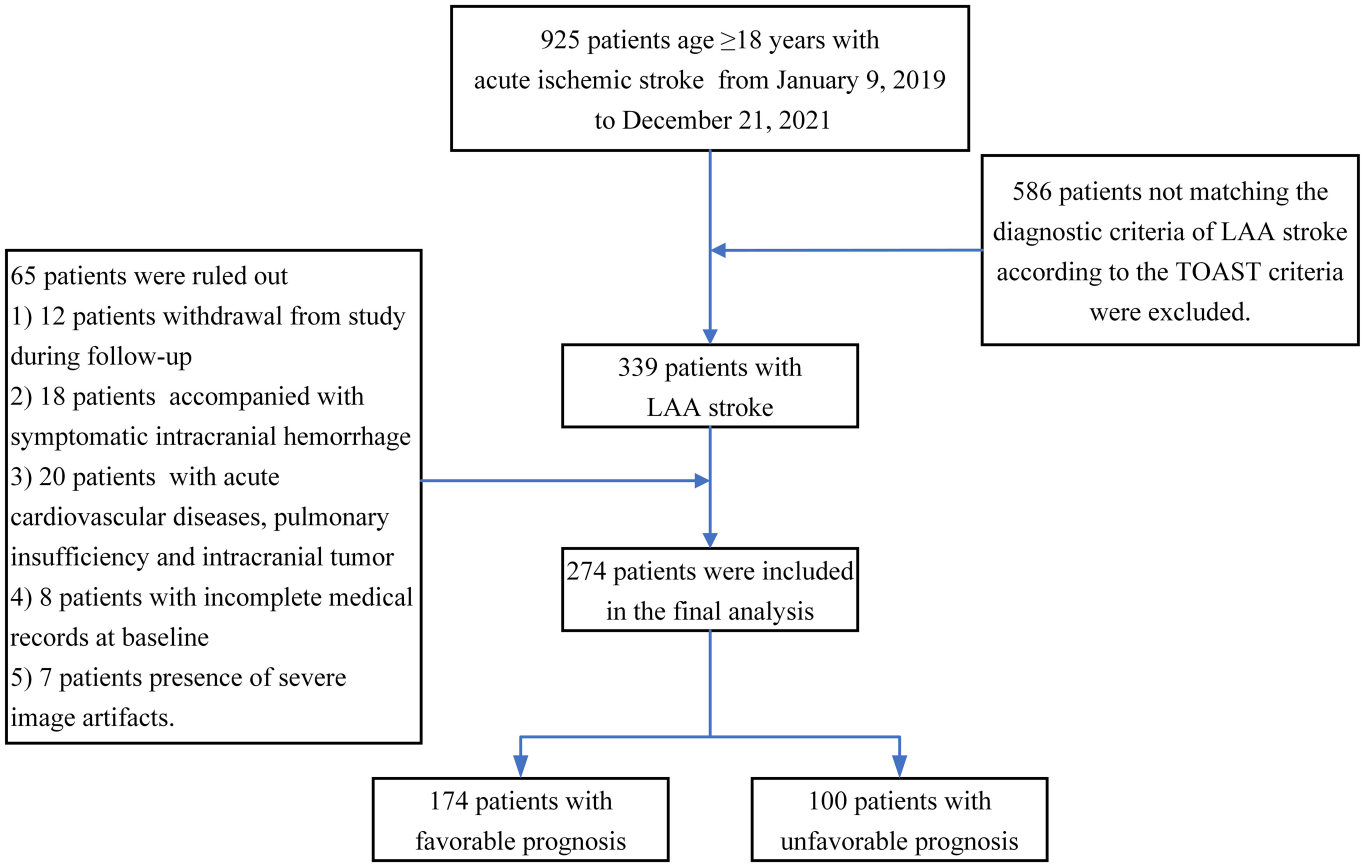
Schematic of the screening process.

This observational, prospective, short-term follow-up and single-center study was conducted on adults with acute LAA stroke. Participants were included if they met all of the following criteria: (1) aged 18 years or older at baseline; (2) MRI and CT angiography (CTA) were performed within 12 h of admission; (3) patients were diagnosed with LAA stroke according to the Trial of Org 10,172 in Acute Stroke Treatment (TOAST) criteria (13): (a) clinical findings include cerebral cortical impairment or brain stem or cerebellar dysfunction; (b) cortical or cerebellar lesions and brainstem or subcortical hemispheric infarcts > 1.5 cm in diameter on CT or MRI; and (c) supportive evidence by duplex imaging of a stenosis of >50% of an appropriate intracranial or extracranial artery; and (4) higher image quality was available for a subsequent neuroimaging evaluation. Patients with poor functional outcomes in the preadmission state (mRS scores of 3–6), acute intracranial hemorrhage, acute cardiovascular diseases, pulmonary insufficiency and intracranial tumors were excluded. A total of 925 patients aged 18 years or older with ischemic stroke were enrolled in the study. All patients provided informed consent and were enrolled if all inclusion criteria and none of the exclusion criteria were met. At the end of the study, only 274 eligible patients were analyzed, and a detailed study flowchart is shown in Figure 1.

### Prognostic assessment

The primary outcome was the distribution of the mRS scores at 3 months. An mRS score <3 indicated a favorable outcome, while an mRS score >2 indicated an unfavorable outcome (14). Patients were then grouped as having a favorable prognosis (mRS score of 0–2) and an unfavorable prognosis (mRS score of 3–6) at 3 months after stroke onset. Patients with recurrent stroke, death, or symptomatic cerebral hemorrhage were eligible for inclusion and classified as having an unfavorable prognosis. Neurological deficits on admission and discharge were assessed using the National Institute of Health Stroke Scale (NIHSS) score. Neurological improvement was defined as improvement of 4 or more points on the NIHSS or an NIHSS score of 0 at discharge (15). Patients were further grouped into NIHSS (favorable) with neurological improvement and NIHSS (unfavorable) without neurological improvement.

### Neuroimaging evaluation

Enrolled patients underwent a brain MRI examination with a 3.0 T scanner (Philips Medical Systems, the Netherlands) with an 8-channel receiver array head coil. Standardized parameters of the MRI sequences, including T1-weighted, T2-weighted and fluid-attenuated inversion recovery images, were obtained. The burden of SVD was graded as 0–4 based on imaging markers (WMH, lacunes, EPVS and CMBs) on MRI using established criteria (16–18). Briefly, one point represents each of the following phenomena: more than 10 EPVS in basal ganglia, presence of lacuna, periventricular WMH with a Fazekas score of 3 or deep WMH with a Fazekas score of 2 or 3, and the presence of deep CMBs. The total SVD score was calculated by summing the scores for the SVD markers listed above.

CTA examinations of the carotid and intracranial arteries were performed with a 64-slice helical CT scanner (Philips Brilliance 64, Philips Healthcare, Amsterdam, Netherlands). Cerebral collaterals were assessed on CTA by consensus by 2 neuroradiologists using the Tan scale (19): 0, absence of collaterals; (1), collaterals filling ≤ 50% of the occluded territory; (2), collaterals filling > 50% but <100% of the occluded territory; and 3, collaterals filling 100% of the occluded territory.

### Statistics

Continuous data are summarized as the mean values with SDs for data with a normal distribution or the median values with interquartile ranges for data with a skewed distribution. Categorical data are presented as frequencies with proportions. A two-sample *t* test was used to compare continuous data. Categorical data were analyzed using the chi-square test. Logistic multivariate analyses were performed to identify the risk factors for the stroke prognosis. Receiver operating characteristic (ROC) curves were constructed to compare the effects of cerebral collaterals and SVD on predicting the prognosis of LAA stroke, and areas under ROC curves (AUCs) were calculated. All statistical analyses were performed using SPSS 25.0 software (**SPSS, Chicago, IL**).

## Results

Of the 274 enrolled patients, 174 (63.50%) were identified as having a favorable prognosis, and 100 (36.50%) were identified as having an unfavorable prognosis. Patients with a favorable prognosis were younger than those with an unfavorable prognosis (66.53 ± 11.34 vs. 71.87 ± 9.90, y, *P* < 0.01). Patients with a favorable prognosis presented lower homocysteine levels (16.59 ± 7.91 vs. 20.20 ± 10.13 μmol/L, *P* < 0.01), total SVD score (2.17 ± 1.04 vs. 2.64 ± 0.95, *P* < 0.01), mRS (2.41 ± 1.39 vs. 3.57 ± 1.09, *P* < 0.01) score and NIHSS score (4.15 ± 3.90 vs. 7.73 ± 5.39, *P* < 0.01) than patients with an unfavorable prognosis at baseline. Patients with a favorable prognosis had a higher Tan score (1.57 ± 0.70 vs. 1.13 ± 0.80, *P* < 0.01) than control subjects. The details are presented in Table 1.

**Table 1:** Clinical characteristics of patients with and without a favorable prognosis at baseline (*n* = 274).

**Variables**	**Patients with a favorable prognosis (*n* = 174)**	**Patients with an unfavorable prognosis (*n* = 100)**	***P* value**
Age, y, mean ± SD	66.53 ± 11.34	71.87 ± 9.90	<0.01
Male, *n* (%)	113 (64.94)	59 (59.00)	0.33
HR, bpm, mean ± SD	75.25 ± 14.24	76.69 ± 14.71	0.43
Pulse pressure, mmHg, mean ± SD	67.60 ± 18.20	68.54 ± 18.33	0.83
**Medical history**, ***n*** **(%)**			
Hypertension	128 (73.56)	76 (76.00)	0.66
Diabetes	65 (37.36)	31 (31.00)	0.29
Coronary artery disease	15 (8.62)	13 (13.00)	0.25
Previous ischemic stroke	56 (32.18)	43 (43.00)	0.19
Atrial fibrillation	12 (6.90)	14 (14.00)	0.05
Current smoker	45 (25.86)	21 (21.00)	0.37
Current alcohol user	33 (18.97)	13 (13.00)	0.20
**Laboratory findings, mean** **±SD**			
Troponin-I, ng/mL	0.02 ± 0.01	0.02 ± 0.02	0.37
Lp-PLA2, ng/mL	235.18 ± 131.80	273.43 ± 143.07	0.05
TC, mmol/L	4.13 ± 0.98	4.45 ± 4.97	0.41
LDL-C, mmol/L	2.58 ± 0.90	2.59 ± 1.07	0.97
HDL-C, mmol/L	1.02 ± 0.24	1.09 ± 0.85	0.31
TG, mmol/L	1.57 ± 0.96	1.46 ± 0.92	0.38
Lipoprotein (a), mg/L	261.02 ± 241.15	292.84 ± 289.89	0.34
Homocysteine, μmol/L	16.59 ± 7.91	20.20 ± 10.13	<0.01
Creatinine, μmol/L	70.46 ± 32.08	72.82 ± 26.48	0.54
Uric acid, μmol/L	313.78 ± 97.17	327.73 ± 105.60	0.27
**Therapy**, ***n*** **(%)**			
Statin therapy	71 (40.80)	41 (41.00)	0.98
antiplatelet therapy	97 (55.75)	55 (55.00)	0.86
IVT or EVT treatment	17 (9.77)	8 (8.00)	0.65
**Related scales, mean** **±SD**			
Total SVD score	2.17 ± 1.04	2.64 ± 0.95	<0.01
Tan score	1.57 ± 0.70	1.13 ± 0.80	<0.01
mRS score	2.41 ± 1.39	3.57 ± 1.09	<0.01
NIHSS score	4.15 ± 3.90	7.73 ± 5.39	<0.01

*Continuous variables are shown as the mean ± standard deviation (SD), categorical variables are shown as numbers combined with percentage (%).Pulse pressure means the difference between the systolic and diastolic pressures; HR, heart rate; IVT, intravenous thrombolysis; EVT, endovascular treatment; LDL-C indicates low-density lipoprotein cholesterol; HDL-C indicates high-density lipoprotein cholesterol; Lp(a), lipoprotein(a); TG, triglycerides; TC, total cholesterol; Lp-PLA2, lipoprotein associated phospholipase A2; mRS, median modified Rankin Scale; NIHSS, National Institute of Health Stroke Scale*.

**Figure 2:**
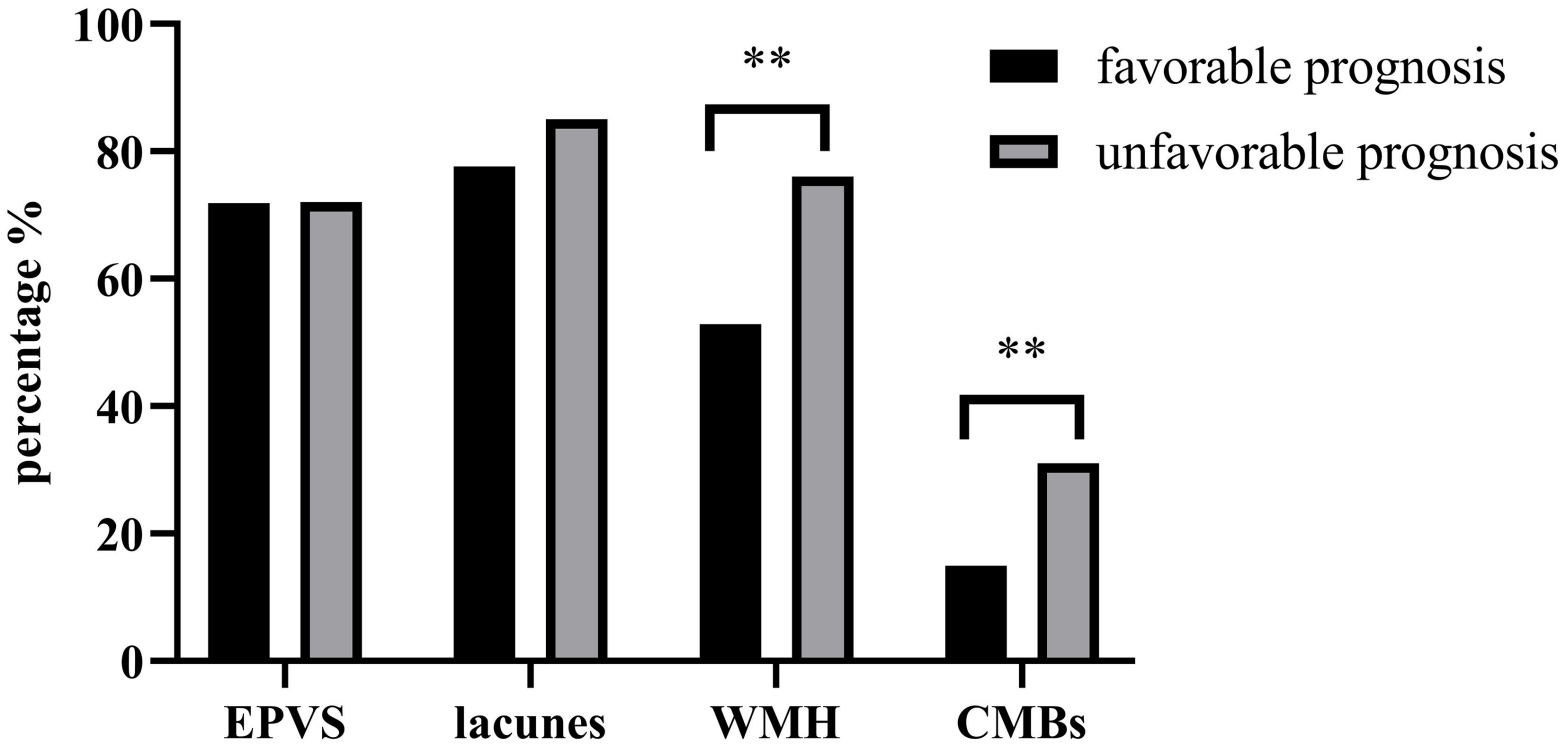
Comparison of EPVS, lacunes, WMH and CMBs between patients with and without a favorable prognosis. ^**^ *P* < 0.01.

We subsequently compared the differences in EPVS, lacunes, WMH and CMBs between patients with and without a favorable prognosis. Patients with a favorable prognosis presented lower ratios of WMH (52.87% vs. 76.00%, *P* < 0.01) and CMBs (14.94% vs. 31.00%, *P* < 0.01) than patients with an unfavorable prognosis, but the ratios of EPVS (71.84% vs. 72.00%, *P* > 0.05) and lacunes (77.59% vs. 85%, *P* > 0.05) were not significantly different between the two groups. This result suggests that the difference in the total SVD burden is mainly derived from WMH and CMBs (Figure 2).

After adjusting for covariates, the logistic regression analysis indicated that an unfavorable prognosis was related to the total SVD score (Model 1, OR = 1.73, 95% CI: 1.15–2.61, *P* < 0.01; Model 2, OR = 1.85, 95% CI: 1.23–2.79, *P* < 0.01) and Tan score (Model 1, OR = 0.38, 95% CI: 0.23–0.64, *P* < 0.01; Model 2, OR = 0.52, 95% CI: 0.33–0.82, *P* < 0.01). Homocysteine levels (Model 1, OR = 1.07, 95% CI: 1.02–1.13, *P* = 0.01; Model 2, OR = 1.06, 95% CI: 1.01–1.12, *P* = 0.02), the mRS score (Model 1, OR = 2.20, 95% CI: 1.60–3.04, *P* < 0.01) and NIHSS score (Model 2, OR = 1.23, 95% CI: 1.12–1.34, *P* < 0.01) were also associated with an unfavorable prognosis (Tables 2, 3).

**Table 2:** The multivariate logistic regression analysis of Model 1.

**Variables**	**β**	**Wals**	**OR (95% CI)**	***P* value**
Age	0.010	0.247	1.01 (0.97–1.05)	0.62
Atrial fibrillation	0.379	0.347	1.46 (0.42–5.14)	0.56
Lp-PLA2 level	0.001	0.006	1.00 (0.99–1.00)	0.94
Homocysteine level	0.066	6.490	1.07 (1.02–1.13)	0.01
Total SVD score	0.549	6.884	1.73 (1.15–2.61)	<0.01
Tan score	−0.959	13.458	0.38 (0.23–0.64)	<0.01
mRS score	0.790	22.995	2.20 (1.60–3.04)	<0.01

*OR, odds ratio; CI, confidence interval. Model 1 adjust for Age, Atrial fibrillation, Lp-PLA2, Homocysteine and mRS score at baseline*.

**Table 3:** The multivariate logistic regression analysis of Model 2.

**Variables**	**β**	**Wals**	**OR (95% CI)**	***P* value**
Age	0.020	0.968	1.02 (0.98–1.06)	0.33
Atrial fibrillation	0.567	0.692	1.76 (0.46–6.72)	0.41
Lp-PLA2 level	0.001	0.004	1.00 (0.99–1.00)	0.95
Homocysteine level	0.060	5.597	1.06 (1.01–1.12)	0.02
Total SVD score	0.616	8.714	1.85 (1.23–2.79)	<0.01
Tan score	−0.659	7.873	0.52 (0.33–0.82)	<0.01
NIHSS score	0.206	20.367	1.23 (1.12–1.34)	<0.01

*OR, odds ratio; CI, confidence interval. Model 2 adjust for Age, Atrial fibrillation, Lp-PLA2, Homocysteine and NIHSS score at baseline*.

**Figure 3:**
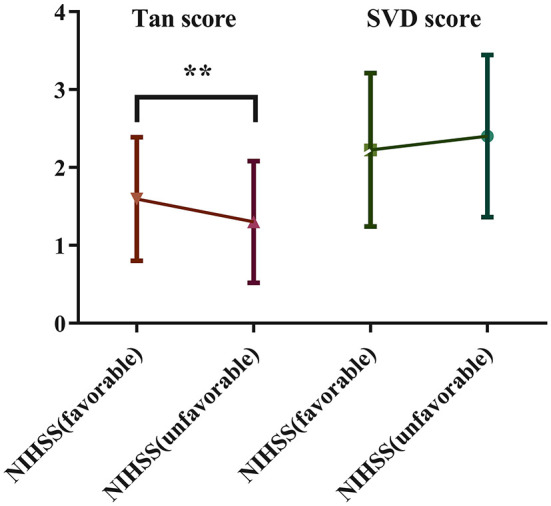
Comparison of the Tan score and SVD score between the two groups stratified based on the NIHSS score.

Patients were stratified according to NIHSS (favorable) and NIHSS (unfavorable) scores. The Tan score and SVD score were compared between the two groups. The results showed that patients in the NIHSS (favorable) group presented significantly higher Tan scores (1.60 ± 0.79 vs. 1.32 ± 0.78, *P* < 0.01) than controls, but the comparison revealed no significant difference in SVD scores (2.23 ± 0.99 vs. 2.39 ± 1.05, *P* = 0.21) between the two groups (Figure 3).

**Figure 4:**
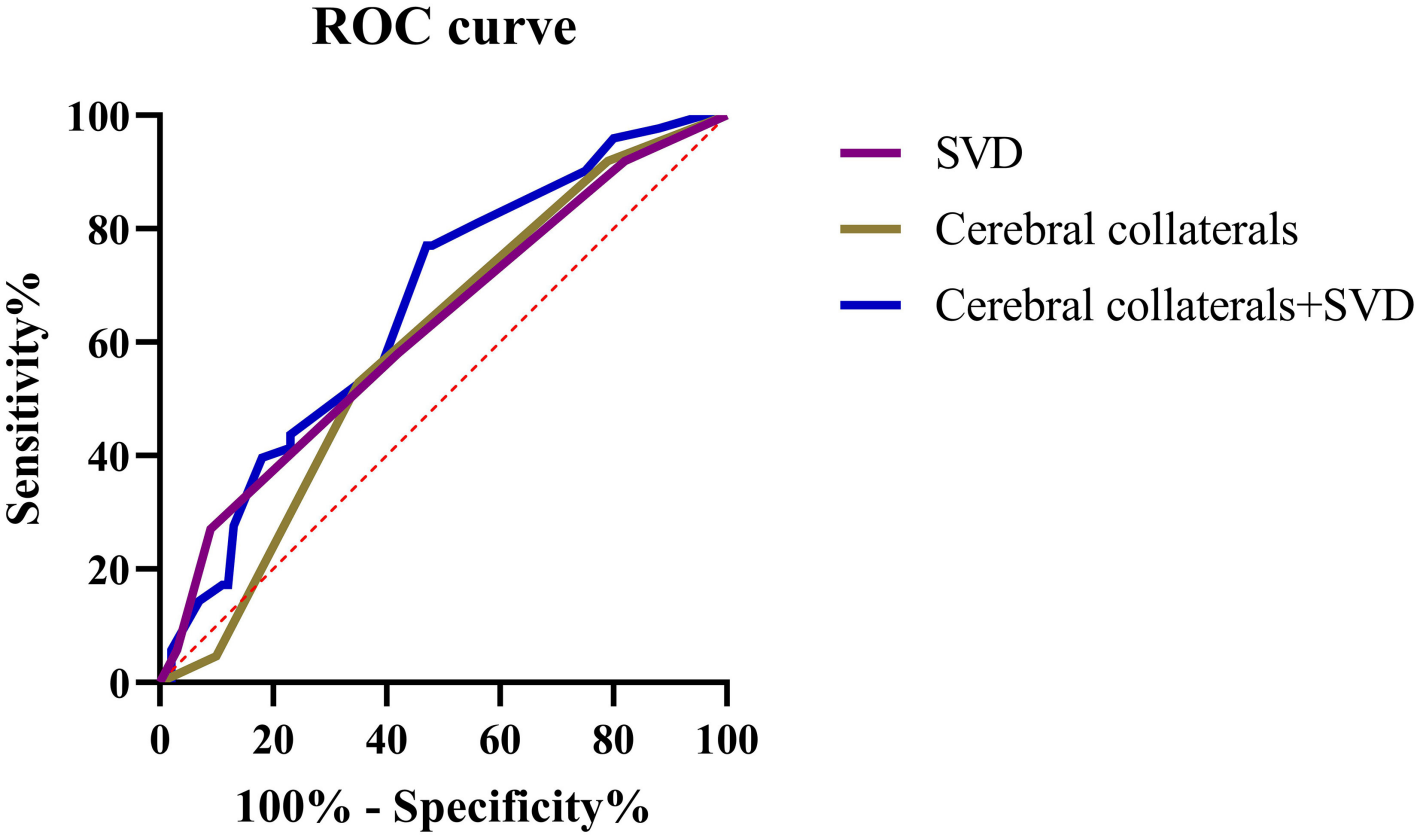
ROC curves for the cerebral collateral circulation and SVD in predicting the unfavorable prognosis of patients with LAA stroke.

ROC curves were created and AUCs were calculated to further evaluate the predictive values of cerebral collateral circulation and SVD in patients with an unfavorable prognosis (Figure 4). Compared with single cerebral collateral circulation (AUC = 0.59; 95% CI: 0.52–0.67; *P* < 0.01) or SVD (AUC = 0.62; 95% CI: 0.56–0.69; *P* < 0.01), the combination of collateral circulation and SVD (AUC = 0.66; 95% CI: 0.59–0.73; *P* < 0.01) has a higher diagnostic value for an unfavorable prognosis, and the optimal sensitivity and specificity were 77.01 and 53.00%, respectively.

## Discussion

In the present study, (1) the total SVD burden was related to the prognosis of patients with LAA stroke, which might primarily arise from the discrepancy of WMH and CMBs. (2) Compared with cerebral collaterals or SVD alone, cerebral collaterals combined with total SVD burden are better at predicting the prognosis of patients with acute LAA stroke. (3) Moreover, cerebral collaterals, not SVD, are associated with poor short-term functional outcomes in patients with LAA stroke.

In general, occlusions of large arteries result from occlusion of the basilar artery, carotid artery and the proximal middle cerebral artery, leading to more serious outcomes, and poorly developed collaterals are often associated with a worse functional prognosis (20). Well-developed collaterals may compensate for acute cerebral hypoperfusion and prolong the time window of intravascular interventional therapy, which is important for extending the therapeutic time window in patients with acute ischemic stroke (21). Good collaterals might reduce the rate of hemorrhagic transformation after thrombolytic or endovascular therapies and the incidence of adverse events (22). Moreover, retrograde collateral flow may help to expose more portions of the thrombus to thrombolytic drugs and promote thrombus dissolution, which is very important in the therapy of acute ischemic stroke (23). Therefore, a good collateral status results in a higher recanalization rate, smaller infarct volume, and better neurological outcome.

Apart from collaterals, a more recent retrospective observational study with similar objective indicated that atrophy and lacune were essential in evaluating stroke patients and could additionally improve the stroke outcome prediction (24). Dissimilarly, our findings suggest that the total burden of SVD was associated with the functional neurological prognosis of patients with LAA stroke, and the difference is mainly attributed to the influence of WMH and CMBs. However, result of the short-term functional outcome based on the changes of NIHSS score at admission and discharge indicated that there was no significant difference in SVD scores between the two groups (Figure 3), though the difference was not statistically significant, patients in the NIHSS (favorable) group exhibited a tendency of lower SVD scores. This is probably due to the relatively short observation time. WMH destroy white matter fiber tracts and the network architecture of the brain, and these changes in white matter tissue microstructure may lead to severe deficits related to impaired brain plasticity (25), potentially resulting in a poor outcome and stroke recurrence (26). Besides, a recent study showed that WMH burden had a dose-dependent relationship with poor collaterals and further led to poor prognosis (27). A randomized trial of large populations showed that WMH was a risk factor for first-ever and recurrent stroke in the general population (28). A study of 307 patients illustrated that the large artery disease group had a higher prevalence of WMH than the other groups (9). Furthermore, several studies have shown that CMBs are associated with hemorrhagic stroke and significantly increase the risk of ischemic stroke, which also substantially affects the neurological function and prognosis of patients with LAA stroke (29, 30). The poor prognosis associated with CMBs may be related to hemorrhagic transformation after ischemic stroke. The results of a multicenter prospective cohort study indicated that patients with multiple CMBs have a six times higher risk of recurrent stroke than those without CMBs and exhibit an increased fatality rate of stroke (31). A population-based cohort study reported that an increase in the mean carotid intima-media thickness, a marker of LAA, was related to an increased risk of CMBs, especially in the deep and infratentorial brain regions (8). EPVS and lacunes may also be related to the prognosis of patients with LAA stroke, although our present study did not observe significant differences in these parameters. EPVS are strongly associated with age and may correlate with the functional outcome and prognosis of patients with LAA stroke. The pathological examination of EPVS revealed that the brain tissue surrounding the lesions was destroyed, accompanied by reactive gliosis, which may contribute to neurological deficits (32). Although lacunes are less severe and have better short-term physical outcomes, patients with the condition are at increased risk of recurrence and neurological impairment over time (33), and lacunes and LAA stroke share common risk factors and influence each other (10). Therefore, more research is needed to prove the correlation between SVD and the prognosis of LAA stroke; nevertheless, research on the mechanism needs to continue.

The current study has several strengths and limitations. First, this study is one of the first to focus on the relationship between the total SVD burden and prognosis of patients with LAA stroke. Second, the study was performed with a short-term follow-up and dynamic observation of progression. Third, we explored the combined effects of SVD burden and cerebral collaterals on the prognosis of patients with acute LAA stroke. Our study is limited by a relatively small sample size and enrollment of patients at a single center, and the study is based on clinical research and does not explore the underlying mechanisms. Moreover, despite the widespread approval of intravenous thrombolysis treatment and endovascular treatment for patients with acute ischemic stroke, the narrow therapeutic windows limit their clinical application, and we did not discuss the effects of different treatments on the outcome. Therefore, further multicenter and in-depth mechanistic studies are needed to overcome the aforementioned limitations.

Taken together, our findings suggest that the total SVD burden was related to the prognosis of patients with LAA stroke, which might primarily arise from the differences in WMH and CMBs. Compared with cerebral collaterals or SVD alone, cerebral collaterals combined with total SVD burden have a better value to predict the prognosis of patients with acute LAA stroke. Moreover, cerebral collaterals, not SVD, are associated with poor short-term functional outcomes of patients with LAA stroke.

## Data availability statement

The original contributions presented in the study are included in the article/supplementary material, further inquiries can be directed to the corresponding author.

## Ethics statement

The studies involving human participants were reviewed and approved by Affiliated Jiangning Hospital with Nanjing Medical University. The patients/participants provided their written informed consent to participate in this study.

## Author contributions

XC contributed to the study design. CW, TS, XT, and XY performed the data collection. XC, CW, TS, and YG were responsible for data analysis and imaging evaluation. CW wrote the manuscript. All authors approved the final manuscript for publication.

## Funding

This research was supported by Jiangning Science and Technology Huimin Project (2022092S), the Nanjing Medical Science and Technique Development Foundation (QRX17032 and YKK20203), the Clinical Medical Science and Technology Development Fund of Jiangsu University (JLY2021153), and the National Nature Science Foundation of China (Grant 81901206).

## Conflict of interest

The authors declare that the research was conducted in the absence of any commercial or financial relationships that could be construed as a potential conflict of interest.

## Publisher's note

All claims expressed in this article are solely those of the authors and do not necessarily represent those of their affiliated organizations, or those of the publisher, the editors and the reviewers. Any product that may be evaluated in this article, or claim that may be made by its manufacturer, is not guaranteed or endorsed by the publisher.

## Abbreviations

LAA: Large artery atherosclerotic
TOAST, Trial of Org 10: 172 in Acute Stroke Treatment
OR: odds ratio
CI: confidence interval
ROC: receiver operating characteristic
AUC: areas under ROC curves
mRS: median modified Rankin Scale
IVT: intravenous thrombolysis
EVT: endovascular treatment
MT: mechanical thrombectomy
MRI: magnetic resonance imaging
CMBs: cerebral microbleeds
WMH: white matter hyperintensities
EPVS: enlarged perivascular spaces
CTA: CT angiography
NIHSS: National Institute of Health Stroke Scale
SVD: small vascular disease
sICH: spontaneous intracerebral hemorrhage
SD: standard deviation
HR: heart rate
LDL-C: indicates low-density lipoprotein cholesterol
HDL-C: indicates high-density lipoprotein cholesterol
Lp(a): lipoprotein(a)
TG: triglycerides
TC: total cholesterol
Lp-PLA2: lipoprotein associated phospholipase *A2*.
